# Low Serum Creatine Kinase Level Predicts Mortality in Patients with a Chronic Kidney Disease

**DOI:** 10.1371/journal.pone.0156433

**Published:** 2016-06-01

**Authors:** Adrien Flahault, Marie Metzger, Jean-François Chassé, Jean-Philippe Haymann, Jean-Jacques Boffa, Martin Flamant, François Vrtovsnik, Pascal Houillier, Bénédicte Stengel, Eric Thervet, Nicolas Pallet, NephroTest study group

**Affiliations:** 1 Service de néphrologie, Hôpital Européen Georges Pompidou, Assistance Publique Hôpitaux de Paris (AP-HP), Paris, France; 2 INSERM UMRS-1018, CESP Team 5 (Renal and Cardiovascular Epidemiology), Villejuif, France; 3 Paris Descartes University, Paris, France, Sorbonne Paris Cité, Paris, France; 4 Clinical chemistry, Hôpital Européen Georges Pompidou, AP-HP, Paris, France; 5 Université Pierre et Marie Curie, INSERM U702, Paris, France; 6 Hôpital Tenon, AP-HP, Service d'Explorations Fonctionnelles, Paris, France; 7 Hôpital Tenon, AP-HP, Service de Néphrologie, Paris, France; 8 Hôpital Bichat, AP-HP, Service de Physiologie, Paris, France; 9 Hôpital Bichat, AP-HP, Service de Néphrologie, Paris, France; 10 Hôpital Européen Georges Pompidou, AP-HP, Service d'Explorations Fonctionnelles, Paris, France; 11 College de France, Laboratory of Central Neuropeptides in the Regulation of Body Fluid Homeostasis and Cardiovascular Functions, Center for Interdisciplinary Research in Biology (CIRB), INSERM U1050, Paris, France; The University of Tokyo, JAPAN

## Abstract

**Background:**

Serum creatine kinase (sCK) reflects CK activity from striated skeletal muscle. Muscle wasting is a risk factor for mortality in patients with chronic kidney disease (CKD). The aim of this study is to evaluate whether sCK is a predictor of mortality and end-stage renal disease (ESRD) in a CKD population.

**Methods:**

We included 1801 non-dialysis-dependent CKD patients from the NephroTest cohort. We used time-fixed and time-dependent cause-specific Cox models to estimate hazard ratios (HRs) for the risk of death and for the risk of ESRD associated with gender-specific sCK tertiles.

**Results:**

Higher sCK level at baseline was associated with a lower age, a higher body mass index, and a higher level of 24 h urinary creatinine excretion, serum albumin and prealbumin (p<0.001). Men, patients of sub-Saharan ancestry, smokers and statin users also experienced a higher level of sCK. In a time-fixed Cox survival model (median follow-up 6.0 years), the lowest gender-specific sCK tertile was associated with a higher risk of death before and after adjustment for confounders (Crude model: hazard ratio (HR) 1.77 (95% CI: 1.34–2.32) compared to the highest tertile; fully-adjusted model: HR 1.37 (95% CI: 1.02–1.86)). Similar results were obtained with a time-dependent Cox model. The sCK level was not associated with the risk of ESRD.

**Conclusion:**

A low level of sCK is associated with an increased risk of death in a CKD population. sCK levels might reflect muscle mass and nutritional status.

## Introduction

Creatine kinase (CK), also referred to as creatine phosphokinase (CPK), is a cytosolic and mitochondrial enzyme that catalyzes the phosphorylation of creatine into phospho-creatinine, a rapidly mobilizable reserve of high energy phosphates: phosphocreatine donates a phosphate group to adenosine diphosphate (ADP) to generate adenosine triphosphate (ATP). CK is an important enzyme in tissues that consumes ATP rapidly, including the muscles and brain [1], and serum CK (sCK) activity measurement is used as a blood test to monitor damage in CK-rich tissues, mainly in striated muscles [2]. sCK activity is currently measured in clinical practice to detect and monitor muscle-associated diseases, including rhabdomyolysis, myocardial infarction, myositis and muscle dystrophy. Beside its diagnostic value of tissue damage, the sCK level yields prognostic value because high blood concentrations of sCK have been associated with increased mortality during rhabdomyolysis [3], traumatic injuries [4], viral infections (for example hantaviruses [5]), and genetic myopathies [6]. In a population of hospitalized, elderly patients, the 1-year mortality rate was high (27%) [7] in individuals with a sCK > 1000 UI/l. A moderately elevated level of sCK was also shown to be associated with an increased risk of high blood pressure [8]. Little is known, however, about its potential role as a marker in muscle wasting.

Muscle wasting is a known risk factor for mortality in dialysis patients [9,10] and occurs early in the history of chronic kidney disease (CKD) [11]. Several studies have associated various biomarkers with muscle mass, including daily urinary creatinine excretion (UCr) [12–14], and a low UCr is associated with increased mortality in CKD patients [15,16]. A limitation to the routine use of UCr in clinical practice is that it requires adequate (neither incomplete nor excessive) 24-h urine collection. Evidence indicates that sCK reflects muscular mass. Indeed, the sCK level is higher in healthy men compared to women [17] and in black individuals compared to Caucasians [18]. sCK has been associated with muscle mass in studies comparing athletes to the general population [19], as well as with body mass index (BMI) [20] and lean body mass [21].

Whether sCK may be a prognostic tool in CKD is currently unknown. To address this question, we undertook a longitudinal population-based observational study on 1801 CKD patients (the NephroTest study) to determine if sCK is associated with the risk of mortality and end stage renal disease (ESRD).

## Materials and Methods

### Study population

**Fig 1:**
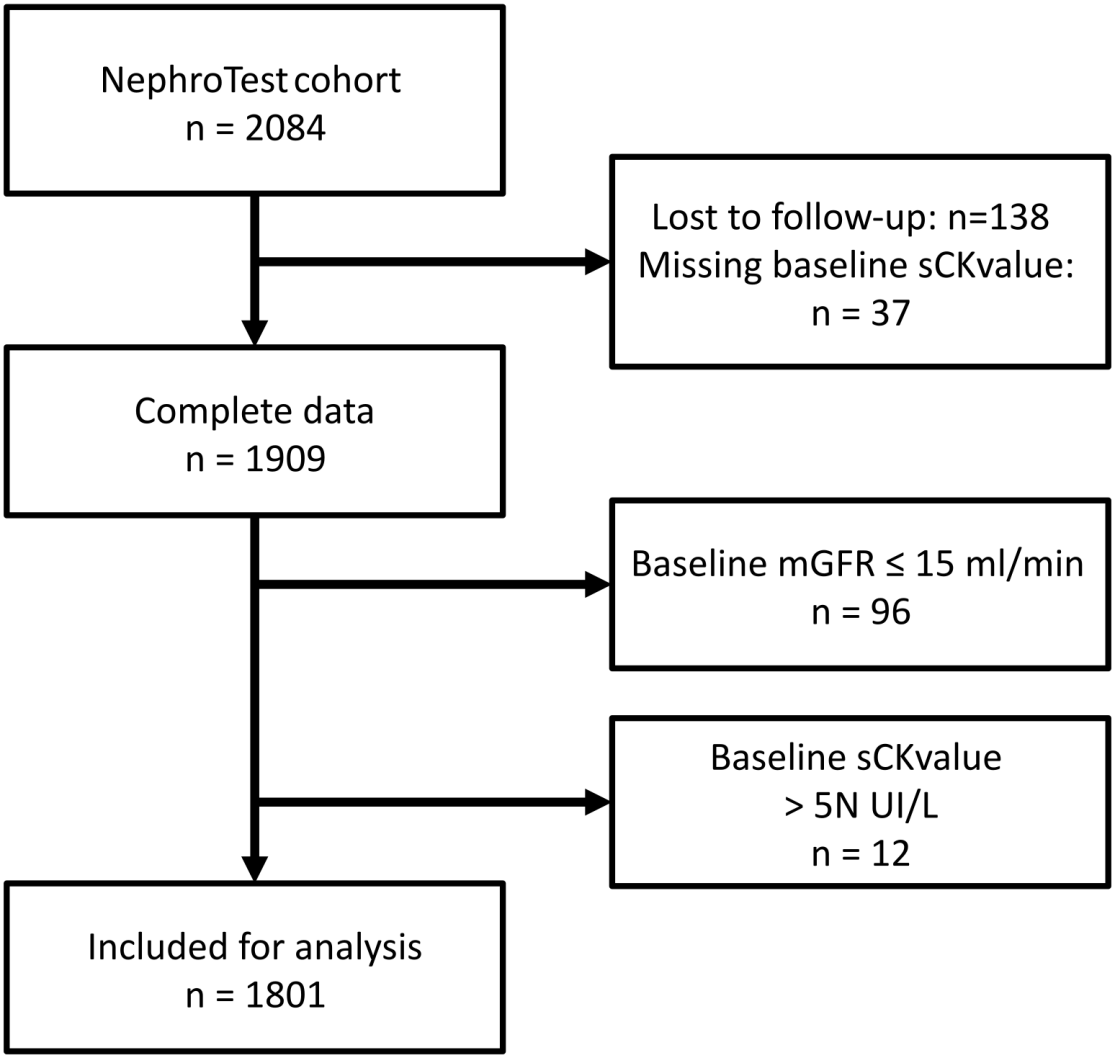
Study flowchart.

The NephroTest study is a multicentric, hospital-based prospective cohort that enrolled 2084 non-ESRD adult patients ≥ 18 years of age between January 2000 and December 2012 with all kidney disease types and all stages of CKD. Patients were enrolled upon discovery of CKD and all patients were referred for GFR measurement at the discretion of the nephrologist. Pregnant women and kidney transplant recipients were excluded. From the initial cohort, 37 patients missed the baseline measurement for sCK levels, and 138 patients were lost to follow-up. Ninety-six (96) patients with a baseline-measured glomerular filtration rate (mGFR) lower than 15 ml/min were excluded. In general, an sCK level >5 times its normal value is accepted by many authors as a diagnostic criteria for rhabdomyolysis [22,23]. Twelve (12) individuals (<1%) with an sCK level > 5 times its normal value (sCK > 855 UI/l in men and > 725 UI/l in women, normal ranges <171 UI/L in males and <145 UI/l in females) were excluded to eliminate cases of patent rhabdomyolysis. Consequently, 1801 patients were included in the present analysis (Fig 1).

### Data collection and outcome

Data at baseline and at each follow-up visit were recorded during a 5-hour in-person visit, which consisted in the collection of a large set of clinical and laboratory indicators, including blood pressure measurement, body mass index (BMI), relevant medical history and treatments received. Follow-up was conducted through December 31, 2013; patients were referred for follow-up GFR measurement at the discretion of their physician, these follow-up visits were not mandatory. Follow-up information concerning vital status was obtained by a linkage with the national death registry. ESRD were collected through medical records and from the French REIN Registry. ESRD was defined by dialysis or preemptive kidney transplantation. All survival data were censored on December 31, 2013 for patients identified from ESRD or death registries or to the date of the last visit.

### Clinical chemistry

Renal clearance of ^51^Cr-EDTA was used to measure GFR at enrollment and each visit, as previously described [24]. In short, a single dose of 1.8–3.5 MBq of ^51^Cr-EDTA (GE Healthcare, Vélizy, France) was injected, and one hour later, the average renal ^51^Cr-EDTA clearance was determined for five to six consecutive 30-minute clearance periods.

The quantitative determination of CK (CK-Nac) is performed using the kinetic measurement of absorbance at 340/660 nm due to the formation of nicotinamide adenine dinucleotide phosphate (NADPH), which is directly proportional to the activity of CK in the sample of human serum. CK-Nac is a modification of the International Federation of Clinical Chemistry (IFCC) methods for the measurements of catalytic concentration of enzymes [25] performed on Beckman Coulter analyzers. Normal ranges are <171 UI/L in males and <145 UI/l in females.

Because a 24-h urine collection may be inaccurate, we made the assumption that a UCr is stable over 24 hours and used a 24-h UCr extrapolated from fractionated creatinine clearance, as described previously [15,26].

### Statistical analysis

Variables were expressed as percentages or medians (interquartile range, IQR). Associations between the baseline sCK level and the covariables were established by comparing 3 groups, defined by their sCK value and expressed as gender-specific tertiles. Comparisons between groups were made using the Kruskal-Wallis test for quantitative covariables and Pearson’s chi-square test for qualitative covariables. Associations between the baseline sCK level were treated as a continuous variable, and the covariables were established as follows: for quantitative measurements, we used Pearson’s correlation coefficients to assess the correlation between the sCK level and continuous variables; for qualitative measurements, we used the Wilcoxon or Kruskal-Wallis tests when appropriate.

To assess the impact of baseline sCK level on the risk of death, the risk of death before ESRD or of ESRD, we conducted cause-specific Cox regression analyses to estimate crude and cause-specific adjusted hazard ratio (HRs) and 95% confidence intervals, with the highest sCK tertile as the reference category. Five nested models allowed for successive adjustment on the following: (1) demographic characteristics (gender, age, ethnicity, medical center), (2) confounding factors (statin intake, ASAT level), (3) mGFR, (4) cardiovascular and renal risk factors (i.e., diabetes, smoking status, systolic blood pressure, history of cardiovascular events, type of nephropathy, logarithm of proteinuria/creatinuria ratio) and finally, (5) markers of nutritional status (BMI, albumin, prealbumin, 24-h creatinuria). Adjusted model 3 was also run with mGFR replaced by GFR estimated (eGFR) using an adjusted CKD-EPI equation [27]. To estimate mortality and ESRD HRs associated with sCK levels during follow-up, we also performed time-dependent Cox models with adjustment covariates similar to the time-fixed models. Subsidiary analyses were performed on sCK treated as a continuous variable. Proportional hazards assumption was verified for each covariate using a two-sided chi-square test of the Schoenfeld residuals.

Because missing values accounted for more than 4% of some covariates (race, prealbumin and 24-hr urinary creatinine), we performed multiple imputations using the MICE method [28], with 20 iterations of 20 imputations. Characteristics in Table 1 were used in the imputation model, as well as used in survival status and time to the censoring event. Missing values for follow-up visits were replaced by the most recent available values. Imputed data sets were analyzed with previously described multivariate Cox survival models, and analysis results were pooled using Rubin and Schencker’s rules [29].

**Table 1:** Clinical and laboratory characteristics at baseline by gender-specific tertile of sCK level.

Characteristic	All	sCK level (gender specific tertile)			p	Missing data n (%)
		1^st^ (lowest)	2^th^	3^th^ (highest)		
Total	1801	610 (34)	596 (33)	595 (33)		
Age (years)	61 (48–71)	64 (52–73)	61 (47–71)	58 (48–69)	< 0.001	0
Male n (%):	1214 (67)	410 (67)	404 (68)	400 (67)	1	0
Female n (%)	587 (33)	200 (33)	192 (32)	195 (33)		
Ethnicity n (%): African origin	224 (13)	11 (2)	44 (8)	169 (30)	< 0.001	81 (4)
Ethnicity n (%): Other	1496 (87)	570 (98)	525 (92)	401 (70)		
Cardiovascular disease n (%): Yes	321 (18)	119 (20)	103 (18)	99 (17)	0.4	37 (2)
Cardiovascular disease n (%): No	1443 (82)	481 (81)	481 (82)	481 (83)		
Diabetes^a^ n (%): Yes	490 (27)	155 (26)	147 (25)	188 (32)	0.01	6 (0.3)
Diabetes^a^ n (%): No	1305 (73)	451 (74)	448 (75)	406 (68)		
Smoking n (%): Non smoker	965 (54)	273 (45)	331 (56)	361 (61)	< 0.001	0
Smoking n (%): Present or past smoker	836 (46)	337 (55)	265 (44)	234 (40)		
BMI < 19 kg/m^2^	61 (3)	28 (5)	24 (4)	9 (2)	< 0.001	0
BMI: 19–25 kg/m^2^	684 (38)	267 (44)	234 (39)	183 (31)		
BMI: 25–30 kg/m^2^	674 (37)	209 (34)	219 (37)	246 (41)		
BMI > 30 kg/m^2^	382 (21)	106 (17)	119 (20)	157 (26)		
Systolic BP (mmHg)	133 (121–147)	133 (121–148)	131 (120–145)	135 (123–148)	0.04	59 (3)
Diastolic BP (mmHg)	74 (67–82)	74 (66–81)	73 (67–81)	75 (68–83)	0.01	59 (3)
Antihypertensive medication n (%): Yes	1586 (92)	518 (89)	523 (91)	545 (95)	0.004	76 (4)
Antihypertensive medication n (%): No	139 (8)	62 (11)	46 (8)	31 (5)		
Statin use n (%): Yes	787 (46)	245 (42)	254 (44)	288 (50)	0.03	76 (4)
Statin use n (%): No	938 (54)	335 (58)	315 (55)	288 (50)		
Serum creatinine (μmol/l)	145 (112–190)	143 (110–188)	142 (109–119)	152 (118–198)	0.005	0
mGFR (ml/min)	40 (29–55)	38 (28–54)	42 (29–55)	41 (30–55)	0.08	0
eGFR (CKD-Epi)	42 (30–58)	41 (30–58)	53 (31–58)	41 (30–57)	0.4	0
PCR (mg/kg)	26 (12–89)	26 (12–80)	23 (11–78)	28 (12–117)	0.3	78 (4)
Albumin (g/l)	40 (37–42)	39 (37–42)	40 (37–42)	40 (37–42)	0.05	43 (2)
Prealbumin (g/l)	0.30 (0.26–0.35)	0.30 (0.26–0.35)	0.30 (0.26–0.35)	0.31 (0.27–0.35)	0.1	384 (21)
ASAT (UI/l)	24 (19–29)	21 (17–26)	24 (19–28)	27 (22–33)	< 0.001	8 (0.4)
HDL-cholesterol (g/l)	1.23 (1.00–1.54)	1.21 (0.96–1.53)	1.25 (1.02–1.56)	1.22 (1.02–1.54)	0.4	71 (4)
UCr (mmol/24h)	12 (9–15)	10.9 (8.8–13.2)	11.7 (9.6–14.2)	12.9 (10.3–15.9)	< 0.001	209 (12)
Nephropathy: PKD	106 (6)	36 (6)	38 (6)	32 (5)	< 0.001	0
Nephropathy: Diabetic	178 (10)	42 (7)	49 (8)	87 (15)		
Nephropathy: Glomerular	259 (14)	84 (14)	92 (15)	83 (14)		
Nephropathy: Interstitial	164 (9)	65 (11)	52 (9)	47 (8)		
Nephropathy: Vascular	464 (26)	161 (26)	151 (25)	152 (26)		
Nephropathy: Other / Undetermined	630 (35)	222 (36)	214 (36)	194 (33)		

CV, cardiovascular; BMI, body mass index; BP, blood pressure; mGFR, measured glomerular filtration rate; PCR, proteinuria/creatinuria ratio; ASAT: Aspartate transaminase, normal range < 35UI/l; Albumin normal range 38–48 g/l; Prealbumin normal range 0.18–0.38 g/l; HDL-cholesterol normal range > 1 mmol/L. PKD: polycystic kidney disease. Results expressed as n (%) for qualitative variables and median (IQR, interquartile range) for quantitative variables.

^a^ Fasting glucose ≥ 7 mmol/L or HbA1c ≥ 6.5 or antidiabetic treatment or reported diabetes.

Statistical analyses were performed using R version 3.2.2 software (R Development Core Team, 2005).

### Ethics statement

All patients gave written informed consent before inclusion in the study. The NephroTest study design was approved by an ethics committee (Direction générale pour la recherche et l’innovation. Comité consultatif sur le traitement de l’information en matière de recherche dans le domaine de la santé (CCTIRS). Réf: DGRI CCTIRS MG/CP09.503, 9th July 2009).

## Results

### Patient Characteristics at baseline and associations with sCK levels

In the study, 1801 individuals were included, and the median baseline sCK level was 122 (IQR = 80–181) UI/L; the median age of participants at inclusion was 61 (48–71) years, and most of the patients were male (67%) (Table 1). Twenty-seven percent of the patients were diabetic. The median mGFR was 40 (29–55) ml/min. Taken as a continuous variable, sCK levels were higher in men (133 (91–199) UI/L in men vs 98 (67–145) in women, p<0.001). Normal laboratory ranges of sCK are different in men and women, and establishing sCK thresholds for high or low values requires differentiating men from women. We therefore used gender-specific tertiles to study sCK, with cutoff points of 106 and 171 UI/l in men and 76 and 124 UI/l in women. A higher sCK tertile was significantly associated with a lower age (p<0.001), high 24-h UCr (p<0.001), and high BMI (p<0.001) but not with mGFR or eGFR. As expected, statin use at inclusion was associated with a higher sCK level (p = 0.03). sCK levels were higher in patients with diabetes and diabetic nephropathy (S1 Table).

### Mortality and ESRD risk according to baseline sCK level

**Fig 2:**
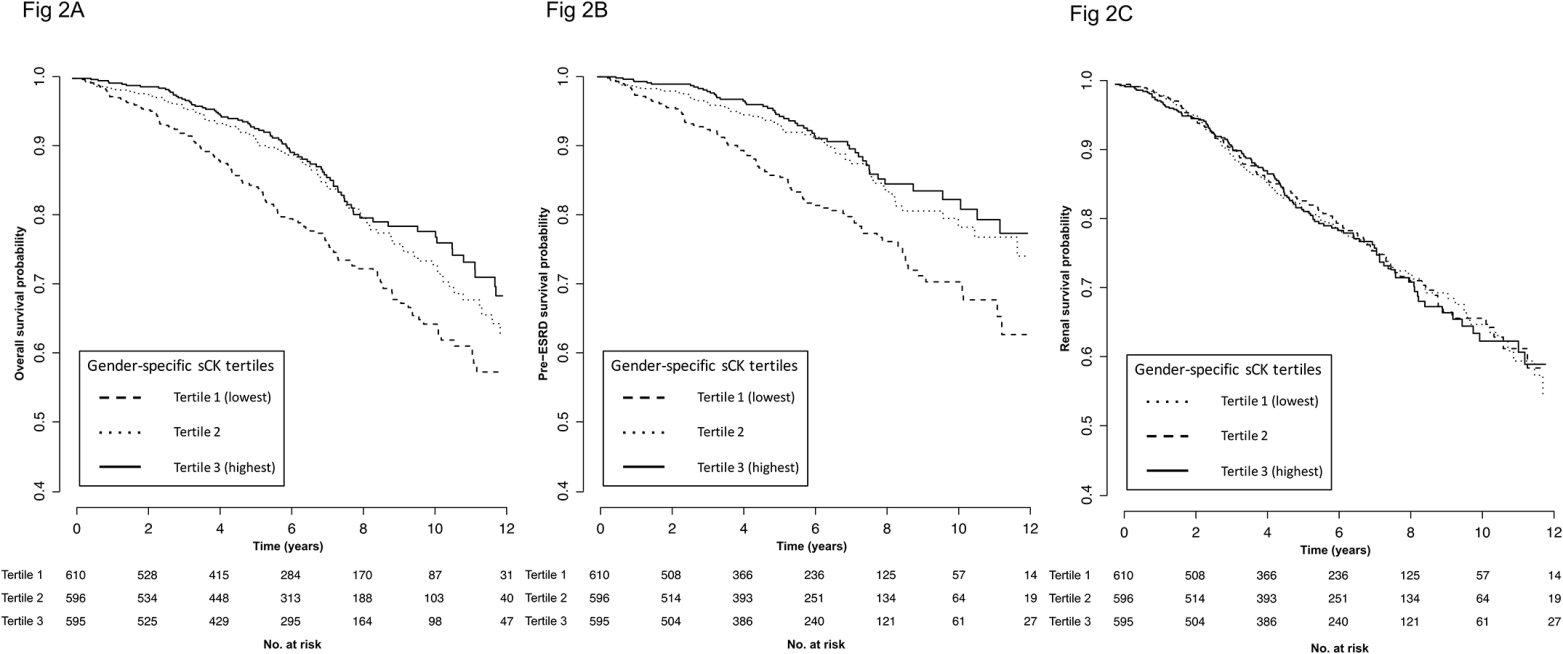
Kaplan-Meier patient survival curves according to gender-specific sCK tertiles. Kaplan-Meier patient overall survival curve according to gender-specific sCK tertiles. Log-rank test: p<0.001. Fig 2B. Kaplan-Meier pre-ESRD patient survival curve according to gender-specific sCK tertiles. Log-rank test: p<0.001. Fig 2C. Kaplan-Meier renal survival curve according to gender-specific sCK tertiles. Log-rank test: p = 0.9.

Over a median follow-up period of 6.0 years (3.6–8.5), 327 (18%) patients died including 221 (12%) before ESRD, and 377 (21%) patients reached ESRD. Patient overall survival and pre-ESRD survival were significantly lower in patients with the lowest baseline sCK level (Fig 2A and 2B). Indeed, patients in the lowest gender-specific sCK tertile had a crude higher risk of death (Hazard Ratio (HR) of 1.77, 95% CI (1.35–2.32) compared to the highest sCK tertile). The lowest sCK tertile was consistently associated with the highest risk of death after adjustment for covariates (Table 2 and S2 Table). In the fully adjusted model (model 5a), using the third (highest) gender-specific sCK tertile as reference HR, the adjusted HR for death (95% CI) was 1.37 (1.02–1.86) for the lowest sCK tertile. In addition to baseline sCK levels, several covariates were independent risk factors for death: age (p<0.001), measured GFR (p<0.001), history of cardiovascular disease (p<0.001), serum albumin (p = 0.04) and prealbumin (p = 0.008), smoking status (p = 0.006) and proteinuria (p = 0.002) (Table 3). Similar results were obtained using pre-ESRD mortality as end-point (S3 Table).

**Table 2:** Crude and fully-adjusted HRs (95% CI) of death according to baseline gender-specific sCK tertiles. The highest gender-specific sCK tertile is taken as the reference tertile.

	gender-specific sCK tertile		
	1^st^ (lowest)	2^nd^	3^rd^ (highest)
Events	142	103	82
Crude	1.77 (1.35–2.32)	1.19 (0.89–1.59)	1
Model 5a	1.37 (1.02–1.86)	1.11 (0.82–1.51)	1

Model 5a: crude + age, gender, ethnicity, center, statin intake, ASAT, measured GFR, history of cardiovascular disease, diabetes, smoking status, systolic blood pressure, type of nephropathy, logarithm of proteinuria/creatinuria ratio, serum albumin, prealbumin, BMI, 24-h urinary creatinine excretion.

**Table 3:** Multivariate adjusted^a^ HRs (95% CI) of baseline covariates for death.

Risk factor	Mortality HR (95% CI)
Gender-specific sCK level	
1^st^ tertile (lowest)	1.37 (1.02–1.86)
2^nd^ tertile	1.11 (0.82–1.51)
3^rd^ tertile (highest)	1
Age, per year greater	1.05 (1.04–1.07)
Women versus men	0.73 (0.52–1.01)
African origin versus other	0.75 (0.43–1.28)
Statin use	0.97 (0.77–1.24)
ASAT, per 1 UI/l	1.01 (1.00–1.02)
mGFR at baseline, per 1 ml/min decrease	1.02 (1.01–1.03)
History of CV disease	1.79 (1.40–2.30)
Diabetes^b^	1.17 (0.87–1.56)
Present or past smoker versus non smoker	1.39 (1.10–1.77)
Systolic BP, per 10 mmHg greater	1.00 (0.99–1.00)
Logarithm of PCR	1.17 (1.06–1.30)
Nephropathy	
PKD	1
Diabetic	1.59 (0.74–3.42)
Glomerular	0.60 (0.26–1.36)
Interstitial	0.79 (0.35–1.77)
Vascular	1.38 (0.69–2.77)
Other / Undetermined	1.39 (1.09–1.77)
Prealbumin, ≤ 0.3 versus > 0.3 g/L	1.44 (1.10–1.88)
24-h urinary creatinine excretion, per mmol/24 h	0.98 (0.94–1.02)

CV, cardiovascular; BMI, body mass index; BP, blood pressure; mGFR, measured glomerular filtration rate; eGFR, estimated glomerular filtration rate; PCR, proteinuria/creatinuria ratio. PKD, polycystic kidney disease. 24-h urinary creatinine excretion is expressed as gender-specific quartiles.

^a^ Including adjustment for center.

^b^ Fasting glucose ≥ 7 mmol/L or HbA1c ≥ 6.5 or antidiabetic treatment or reported diabetes

The Kaplan-Meier curve indicates that the incidence of ESRD is comparable between the three sCK tertiles, and the crude HR (95% IC) is 0.98 (0.76–1.26), 1.01 (0.79–1.29) and 1 for the 1^st^, 2^nd^ and 3^rd^ tertiles, respectively (Fig 2C).

### Mortality risk associated with sCK levels over time

Given that sCK may fluctuate over time, we used a time-dependent Cox model to evaluate the crude and adjusted HRs (Table 4 and S4 Table) of mortality associated with sCK levels at each patient visit. In the crude model, mortality risk was significantly higher in the lowest gender-specific sCK tertile compared with the third (highest) sCK tertile: HR (95% IC) 2.13 (1.63–2.77). These results were similar in the different adjusted models; in the fully adjusted model (model 5a), using the third (highest) gender-specific sCK tertile as a reference HR, the adjusted HR for death (95% CI) was 2.17 (1.51–3.12) for the lowest sCK tertile.

**Table 4:** Crude and fully-adjusted HRs (95% CI) of death according to time-dependent gender-specific sCK tertiles.

	1^st^ (lowest)	2^th^	3^th^ (highest)
Crude	2.13 (1.63–2.77)	1.09 (0.80–1.48)	1
Model 5a	1.74 (1.31–2.32)	1.00 (0.73–1.37)	1

Model 5a: crude + age, gender, ethnicity, center, statin intake, ASAT, measured GFR, history of cardiovascular disease, diabetes, smoking status, systolic blood pressure, type of nephropathy, logarithm of proteinuria/creatinuria ratio, serum albumin, prealbumin, BMI, 24-h urinary creatinine excretion.

## Discussion

Our results indicate that the sCK level is an independent predictor of mortality but not ESRD in patients with CKD. Furthermore, our data support the hypothesis that the sCK level can be used as a proxy for muscle mass evaluation.

We excluded 12 patients with an sCK level higher than five times the normal level, as this is the usual biologic threshold for sCK levels to support rhabdomyolysis. High sCK levels at baseline were not associated with ESRD risk, suggesting that moderately elevated sCK might not be accompanied by renal tubular toxicity and incident renal failure that can occur upon rhabdomyolysis.

Rather, we showed that low sCK levels, both at baseline and over the follow-up period, were significantly associated with mortality in CKD patients, independent of confounding factors. This led us to conclude that sCK is an independent and strong risk factor of mortality among CKD patients.

Because sCK levels were positively correlated with numerous parameters that more or less directly reflect muscle mass, including younger age, BMI, ethnicity, and UCr, a known marker of muscle mass [30], we propose that sCK levels reflect muscle mass and that patients with low muscle mass and a low sCK level experience higher mortality rates. Indeed, nutritional status, including catabolic versus anabolic rates, is a strong predictor of all-cause mortality [31]. Supporting the link between sCK and muscle mass, clinical data indicate that sCK activity is related to lean body mass [21] and that sCK levels at rest are higher in athletes compared to sedentary subjects, possibly due to the regular training athletes undergo [32,33], and, consequently, result in increased anabolism [19,34]. Another possible explanation for these results is that sCK level reflects physical activity beyond its impact on muscle mass. Indeed, intense or prolonged physical activity is associated with a physiological elevation of sCK levels [35,36]. Participants were not asked to rest prior to blood sampling, and we cannot exclude that sCK levels can be influenced by recent physical activity, at least in some individuals. Notably, physical inactivity has been associated with mortality in both CKD and non-CKD populations [37].

We also found a significant association between sCK levels and nutritional markers such as BMI, serum albumin and prealbumin, but this association was not as strong as with UCr. In fact, the median serum albumin and prealbumin values were almost equal in all sCK tertiles. It is therefore likely that sCK is a better predictor of muscle mass than serum albumin and prealbumin. Furthermore, serum albumin is difficult to interpret in the context of nephropathy due to albumin urinary loss. sCK was also positively associated with serum creatinine. Serum creatinine is increased in the presence of renal failure but is also a marker of muscle mass [38], which could explain this correlation.

Notably, we are unable to provide direct markers of muscle mass to confirm the role of sCK as a biomarker for muscle mass. The strong correlation of sCK to surrogate markers, such as creatinine and UCr, as well as converging data in the literature, is, however, very suggestive of a strong association between sCK and muscle mass.

This study only included pre-ESRD CKD patients with a median GFR of 40 ml/min. Although sCK levels have been reported to be associated with nutrition status and muscle mass in non-CKD populations, no study has shown, to this date, a relationship between sCK levels and mortality in the general population. The impact of low sCK levels might therefore differ in other populations.

In conclusion, this study is the first to establish the role of sCK as an independent predictor of mortality in a CKD population. Our results indicate that sCK might reflect nutritional status, and could also be used as a biomarker for muscle mass gain or loss in this population.

## Supporting Information

S1 Table sCK levels according to clinical characteristics at baseline. Categorical covariables expressed as median CK level (interquartile range), significance established using the p-value of a Wilcoxon test (2 categories) or Kruskal-Wallis test followed by Dunn’s test with Holm’s correction when appropriate (>2 categories). Continuous covariables expressed as correlation coefficient, significance established using the p-value of a Pearson’s test. CV, cardiovascular; BMI, body mass index; BP, blood pressure; mGFR, measured glomerular filtration rate; PCR, proteinuria/creatinuria ratio.
(DOCX)

S2 Table Crude and adjusted HRs (95% CI) of death according to baseline gender-specific sCK tertiles. The highest gender-specific sCK tertile is taken as the reference tertile. Model 1: crude + age, gender, ethnicity, center. Model 2: Model 1 + statin intake, ASAT. Model 3a: Model 2 + measured GFR. Model 3b: Model 2 + estimated GFR (CDK-EPI). Model 4: Model 3 + history of cardiovascular disease, diabetes, smoking status, systolic blood pressure, type of nephropathy, logarithm of proteinuria/creatinuria ratio. Model 5a: Model 4 + serum albumin, prealbumin, BMI, 24-h urinary creatinine excretion. Model 5b: Model 5a with estimated GFR instead of measured GFR.
(DOCX)

S3 Table Crude and adjusted HRs (95% CI) of death before ESRD according to baseline gender-specific sCK tertiles. The highest gender-specific sCK tertile is taken as the reference tertile. Model 1: crude + age, gender, ethnicity, center. Model 2: Model 1 + statin intake, ASAT. Model 3a: Model 2 + measured GFR. Model 3b: Model 2 + estimated GFR (CDK-EPI). Model 4: Model 3 + history of cardiovascular disease, diabetes, smoking status, systolic blood pressure, type of nephropathy, logarithm of proteinuria/creatinuria ratio. Model 5a: Model 4 + serum albumin, prealbumin, BMI, 24-h urinary creatinine excretion. Model 5b: Model 5a with estimated GFR instead of measured GFR.
(DOCX)

S4 Table Crude and adjusted HRs (95% CI) of death according to time-dependent gender-specific sCK tertiles. Model 1: crude + age, gender, ethnicity, center. Model 2: Model 1 + statin intake, ASAT. Model 3a: Model 2 + measured GFR. Model 3b: Model 2 + estimated GFR (CDK-EPI). Model 4: Model 3 + history of cardiovascular disease, diabetes, smoking status, systolic blood pressure, type of nephropathy, logarithm of proteinuria/creatinuria ratio. Model 5a: Model 4 + serum albumin, prealbumin, BMI, 24-h urinary creatinine excretion. Model 5b: Model 5a with estimated GFR instead of measured GFR.
(DOCX)
