# Supplementary material for: Low Serum Creatine Kinase Level Predicts Mortality in Patients with a Chronic Kidney Disease
Source: PLoS One. 2016 Jun 1;11(6):e0156433. doi: 10.1371/journal.pone.0156433 (PMC4889148; doi:10.1371/journal.pone.0156433)
Supplement: S1 Table — Categorical covariables expressed as median CK level (interquartile range), significance established using the p-value of a Wilcoxon test (2 categories) or Kruskal-Wallis test followed by Dunn’s test with Holm’s correction when appropriate (>2 categories). Continuous covariables expressed as correlation coefficient, significance established using the p-value of a Pearson’s test. CV, cardiovascular; BMI, body mass index; BP, blood pressure; mGFR, measured glomerular filtration rate; PCR, proteinuria/creatinuria ratio. (DOCX) [file pone.0156433.s001.docx]

**S1 Table. sCK levels according to clinical characteristics at baseline.**

| Characteristic | sCK UI/l (IQR) | Correlation  coefficient | *p* |
| --- | --- | --- | --- |
|  |  |  |  |
| Age |  | -0.11 | < 0.001 |
| Male | 133 (91-199) |  | < 0.001 |
| Female | 98 (67-145) |  |  |
| African origin | 210 (161-319) |  | < 0.001 |
| Ethnicity: Other | 113 (75-163) |  |  |
| Cardiovascular disease: Yes | 126 (79-179) |  | 0.9 |
| Cardiovascular disease: No | 121 (80-182) |  |  |
| Diabetes^a^: Yes | 136 (85-199) |  | < 0.001 |
| Diabetes^a^: No | 118 (77-174) |  |  |
| Smoking: Non smoker | 117 (76-171) |  | < 0.001 |
| Smoking: Present or past smoker | 126 (84-192) |  |  |
| BMI < 19 kg/m^2^ | 95 (58-125) |  | 1 |
| BMI: 19-25 kg/m^2^ | 110 (73-160) |  | 0.03 |
| BMI: 25-30 kg/m^2^ | 131 (85-198) |  | < 0.001 |
| BMI > 30 kg/m^2^ | 140 (93-205) |  | < 0.001 |
| Systolic BP |  | 0.06 | 0.02 |
| Diastolic BP |  | 0.09 | < 0.001 |
| Antihypertensive medication: Yes | 124 (82-187) |  | < 0.001 |
| Antihypertensive medication: No | 101 (66-141) |  |  |
| Statin use: Yes | 131 (84-192) |  | < 0.001 |
| Statin use: No | 115 (77-171) |  |  |
| Serum creatinine |  | 0.12 | < 0.001 |
| mGFR |  | 0.06 | 0.006 |
| eGFR |  | 0.003 | 0.9 |
| PCR |  | 0.02 | 0.38 |
| Albumin |  | 0.09 | < 0.001 |
| Prealbumin |  | 0.12 | < 0.001 |
| ASAT |  | 0.32 | < 0.001 |
| HDL-cholesterol |  | -0.06 | 0.01 |
| UCr |  | 0.36 | < 0.001 |
| Nephropathy: PKD | 116 (82-163) |  | 1 |
| Nephropathy: Diabetic | 160 (102-227) |  | < 0.001 |
| Nephropathy: Glomerular | 124 (82-181) |  | 0.7 |
| Nephropathy: Interstitial | 103 (66-155) |  | 0.4 |
| Nephropathy: Vascular | 122 (80-187) |  | 0.6 |
| Nephropathy: Other / Undetermined | 116 (78-174) |  | 0.6 |

Categorical covariables expressed as median CK level (interquartile range), significance established using the p-value of a Wilcoxon test (2 categories) or Kruskal-Wallis test followed by Dunn’s test with Holm’s correction when appropriate (>2 categories). Continuous covariables expressed as correlation coefficient, significance established using the p-value of a Pearson’s test. CV, cardiovascular; BMI, body mass index; BP, blood pressure; mGFR, measured glomerular filtration rate; PCR, proteinuria/creatinuria ratio. PKD: Polycystic kidney disease
