# Supplementary material for: Low Serum Creatine Kinase Level Predicts Mortality in Patients with a Chronic Kidney Disease
Source: PLoS One. 2016 Jun 1;11(6):e0156433. doi: 10.1371/journal.pone.0156433 (PMC4889148; doi:10.1371/journal.pone.0156433)
Supplement: S2 Table — The highest gender-specific sCK tertile is taken as the reference tertile. Model 1: crude + age, gender, ethnicity, center. Model 2: Model 1 + statin intake, ASAT. Model 3a: Model 2 + measured GFR. Model 3b: Model 2 + estimated GFR (CDK-EPI). Model 4: Model 3 + history of cardiovascular disease, diabetes, smoking status, systolic blood pressure, type of nephropathy, logarithm of proteinuria/creatinuria ratio. Model 5a: Model 4 + serum albumin, prealbumin, BMI, 24-h urinary creatinine excretion. Model 5b: Model 5a with estimated GFR instead of measured GFR. (DOCX) [file pone.0156433.s002.docx]

**Table S2**. Crude and adjusted HRs (95% CI) of death according to baseline gender-specific sCK tertiles. The highest gender-specific sCK tertile is taken as the reference tertile.

|  | **gender-specific sCK tertile** | | |
| --- | --- | --- | --- |
|  | 1^st^  (lowest) | 2^nd^ | 3^rd^  (highest) |
| Events | 142 | 103 | 82 |
| Crude | 1.77 (1.35-2.32) | 1.19 (0.89-1.59) | 1 |
| Model 1 | 1.33 (1.01-1.75) | 1.06 (0.80-1.42) | 1 |
| Model 2 | 1.38 (1.03-1.84) | 1.06 (0.78-1.43) | 1 |
| Model 3a | 1.29 (0.96-1.73) | 1.04 (0.77-1.40) | 1 |
| Model 3b | 1.41 (1.05-1.89) | 1.09 (0.80-1.47) | 1 |
| Model 4 | 1.46 (1.09-1.96) | 1.13 (0.84-1.53) | 1 |
| Model 5a | 1.37 (1.02-1.86) | 1.11 (0.82-1.51) | 1 |
| Model 5b | 1.41 (1.04-1.91) | 1.14 (0.84-1.55) | 1 |

Model 1: crude + age, gender, ethnicity, center.

Model 2: Model 1 + statin intake, ASAT.

Model 3a: Model 2 + measured GFR.

Model 3b: Model 2 + estimated GFR (CDK-EPI).

Model 4: Model 3 + history of cardiovascular disease, diabetes, smoking status, systolic blood pressure, type of nephropathy, logarithm of proteinuria/creatinuria ratio.

Model 5a: Model 4 + serum albumin, prealbumin, BMI, 24-h urinary creatinine excretion.

Model 5b: Model 5a with estimated GFR instead of measured GFR.
