# Supplementary material for: Low Serum Creatine Kinase Level Predicts Mortality in Patients with a Chronic Kidney Disease
Source: PLoS One. 2016 Jun 1;11(6):e0156433. doi: 10.1371/journal.pone.0156433 (PMC4889148; doi:10.1371/journal.pone.0156433)
Supplement: S3 Table — The highest gender-specific sCK tertile is taken as the reference tertile. Model 1: crude + age, gender, ethnicity, center. Model 2: Model 1 + statin intake, ASAT. Model 3a: Model 2 + measured GFR. Model 3b: Model 2 + estimated GFR (CDK-EPI). Model 4: Model 3 + history of cardiovascular disease, diabetes, smoking status, systolic blood pressure, type of nephropathy, logarithm of proteinuria/creatinuria ratio. Model 5a: Model 4 + serum albumin, prealbumin, BMI, 24-h urinary creatinine excretion. Model 5b: Model 5a with estimated GFR instead of measured GFR. (DOCX) [file pone.0156433.s003.docx]

**Table S3**. Crude and adjusted HRs (95% CI) of death before ESRD according to baseline gender-specific sCK tertiles. The highest gender-specific sCK tertile is taken as the reference tertile.

|  | **gender-specific sCK tertile** | | |
| --- | --- | --- | --- |
|  | 1^st^  (lowest) | 2^th^ | 3^th^  (highest) |
| Events | 106 | 64 | 50 |
| Crude | 2.15 (1.54-3.01) | 1.24 (0.85-1.79) | 1 |
| Model 1 | 1.59 (1.13-2.23) | 1.07 (0.73-1.55) | 1 |
| Model 2 | 1.55 (1.09-2.22) | 1.01 (0.69-1.48) | 1 |
| Model 3a | 1.46 (1.03-2.09) | 1.00 (0.68-1.46) | 1 |
| Model 3b | 1.58 (1.11-2.25) | 1.03 (0.70-1.51) | 1 |
| Model 4 | 1.70 (1.18-2.43) | 1.08 (0.73-1.58) | 1 |
| Model 5a | 1.52 (1.05-2.19) | 1.00 (0.68-1.47) | 1 |
| Model 5b | 1.57 (1.09-2.27) | 1.05 (0.71-1.54) | 1 |

Model 1: crude + age, gender, ethnicity, center.

Model 2: Model 1 + statin intake, ASAT.

Model 3a: Model 2 + measured GFR.

Model 3b: Model 2 + estimated GFR (CDK-EPI).

Model 4: Model 3 + history of cardiovascular disease, diabetes, smoking status, systolic blood pressure, type of nephropathy, logarithm of proteinuria/creatinuria ratio.

Model 5a: Model 4 + serum albumin, prealbumin, BMI, 24-h urinary creatinine excretion.

Model 5b: Model 5a with estimated GFR instead of measured GFR.
