# Supplementary material for: Low Serum Creatine Kinase Level Predicts Mortality in Patients with a Chronic Kidney Disease
Source: PLoS One. 2016 Jun 1;11(6):e0156433. doi: 10.1371/journal.pone.0156433 (PMC4889148; doi:10.1371/journal.pone.0156433)
Supplement: S4 Table — Model 1: crude + age, gender, ethnicity, center. Model 2: Model 1 + statin intake, ASAT. Model 3a: Model 2 + measured GFR. Model 3b: Model 2 + estimated GFR (CDK-EPI). Model 4: Model 3 + history of cardiovascular disease, diabetes, smoking status, systolic blood pressure, type of nephropathy, logarithm of proteinuria/creatinuria ratio. Model 5a: Model 4 + serum albumin, prealbumin, BMI, 24-h urinary creatinine excretion. Model 5b: Model 5a with estimated GFR instead of measured GFR. (DOCX) [file pone.0156433.s004.docx]

**Table S4**. Crude and adjusted HRs (95% CI) of death according to time-dependent gender-specific sCK tertiles.

|  | 1^st^  (lowest) | 2^th^ | 3^th^  (highest) |
| --- | --- | --- | --- |
| Crude | 2.13 (1.63-2.77) | 1.09 (0.80-1.48) | 1 |
| Model 1 | 1.59 (1.22-2.08) | 0.98 (0.72-1.34) | 1 |
| Model 2 | 1.69 (1.28-2.23) | 0.99 (0.72-1.36) | 1 |
| Model 3a | 1.60 (1.21-2.11) | 0.96 (0.70-1.32) | 1 |
| Model 3b | 1.69 (1.28-2.23) | 0.97 (0.71-1.33) | 1 |
| Model 4 | 1.78 (1.35-2.36) | 1.01 (0.74-1.38) | 1 |
| Model 5a | 1.74 (1.31-2.32) | 1.00 (0.73-1.37) | 1 |
| Model 5b | 1.79 (1.34-2.37) | 1.00 (0.73-1.37) | 1 |

Model 1: crude + age, gender, ethnicity, center.

Model 2: Model 1 + statin intake, ASAT.

Model 3a: Model 2 + measured GFR.

Model 3b: Model 2 + estimated GFR (CDK-EPI).

Model 4: Model 3 + history of cardiovascular disease, diabetes, smoking status, systolic blood pressure, type of nephropathy, logarithm of proteinuria/creatinuria ratio

Model 5a: Model 4 + serum albumin, prealbumin, BMI, 24-h urinary creatinine excretion.

Model 5b: Model 5a with estimated GFR instead of measured GFR.
